# The Efficacy of the Combination of Continuous Femoral Nerve Block and Intravenous Parecoxib on Rehabilitation in Patients Undergoing Total Knee Arthroplasty: A Double-Blind, Randomized Clinical Trial

**DOI:** 10.7759/cureus.56420

**Published:** 2024-03-18

**Authors:** Despoina Sarridou, Anna Gkiouliava, Helena Argiriadou, Giustino Varrassi, Georgia Chalmouki, Athina Vadalouca, Eleni Moka

**Affiliations:** 1 Anesthesiology and Intensive Care, School of Medicine, Aristotle University of Thessaloniki, Thessaloniki, GRC; 2 Pain Medicine, Paolo Procacci Foundation, Rome, ITA; 3 Anesthesiology, Asklepieion Voulas General Hospital, Athens, GRC; 4 Pain and Palliative Care, Athens Medical Center, Athens, GRC; 5 Anesthesiology, Creta InterClinic Hospital, Herakleion, GRC

**Keywords:** postoperative morphine use, intravenous patient-controlled analgesia, postoperative analgesia, range of motion (rom), rehabilitation, femoral block, parecoxib, total knee arthroplasty (tka)

## Abstract

Background and aim: The optimal strategy for the management of postoperative pain after total knee arthroplasty (TKA) remains challenging, while its treatment is crucial to increase patients’ outcomes. This study aimed to investigate the effects of parecoxib as add-on therapy, in a standard postoperative pain management protocol, represented by the continuous femoral nervous block. We studied its influence on rehabilitation indices and pain scores in patients undergoing TKA.

Material and methods: This is a single-center, prospective, double-blind, randomized, placebo-controlled trial. All patients were operated with the use of subarachnoid anesthesia, and divided into two groups for postoperative analgesia. Both groups received a continuous femoral nerve block. One of the groups received intravenous parecoxib, while the other received a placebo. The primary investigated outcome was the range of motion (ROM). Recordings were noted at different times postoperatively. Bromage score (BS), visual analog scale (VAS), and the State-Trait Anxiety Inventory (STAI) were also studied.

Results: A total of 90 patients were included and analyzed. ROM was significantly better (p<0.001) and pain scores were significantly lower (p=0.007) in the parecoxib group. No statistically significant difference was found with regard to BS between the two groups. A significant correlation was found between ROM and VAS pain scores at 12 hours (p=0.02), while ROM was inversely correlated with STAI postoperatively.

Conclusions: The use of intravenous parecoxib is effective in improving rehabilitation indices and provides decreased postoperative pain scores after TKA.

## Introduction

Total knee arthroplasty (TKA) is the indicated treatment for advanced degenerative joint problems as it can help in relieving pain, restoring joint function, and optimizing health and living standards [1,2]. Nonetheless, it requires an effective postoperative analgesia plan, as TKA is considered one of the most painful procedures in orthopedic surgery [3-5]. As much as 50% of TKA patients report severe pain after surgery, leading to prolongation of the rehabilitation period, reduced cooperation during physiotherapy sessions, and increased risk of chronic postsurgical pain development [6-10]. The aforementioned aspects contribute to longer hospital stays, higher costs, and an adverse socioeconomic impact [11,12].

Non-steroidal anti-inflammatory drugs (NSAIDs) are normally administered to decrease opioid use and the related side effects during and after surgery [13]. Surgical bleeding and gastrointestinal complications have been linked to the use of NSAIDs. Cyclooxygenase-2 (COX-2) selective inhibitors have been suggested as a safer alternative [14]. Parecoxib, in particular, reduces opioid intake after TKA [15]. A single dose of 40 mg provides enough analgesic effect to tackle medium to severe post-TKA pain that may be critical for early movement and rehabilitation [16]. This study aimed to investigate the potential influence of combining intravenous parecoxib with continuous femoral nerve block (CFNB) on rehabilitation and length of hospital stay of patients undergoing TKA.

## Materials and methods

Type of study, ethical considerations, and randomization process

This is a single-center, prospective, double-blinded, randomized, placebo-controlled trial, including adult patients undergoing scheduled TKA with the use of subarachnoid anesthesia. Prior to this, in all patients, a catheter was applied under neurostimulation guidance for the CFNB. This block has previously resulted as the most efficacious peripheral nerve block in postoperative rehabilitation in this population [17]. Patients were randomly allocated into the following two groups in relation to the placebo/parecoxib administration: Group D (drug) received parecoxib 40 mg twice per day, with the first dose being administered 20 min prior to surgery completion and for every 12 h within a 48-h period, and Group P (placebo) received the placebo drug (normal saline 0.9%) instead of parecoxib at the same time points. Both were injected intravenously. Table 1 reports the study inclusion and exclusion criteria.

**Table 1:** Inclusion and exclusion criteria of this study.

Inclusion criteria	Exclusion criteria
Age between 40 and 80 years	Obesity (BMI >30 kg/cm^2^)
Scheduled total knee arthroplasty (TKA)	Previous use of aspirin
Provision of written informed consent	Known allergy to local anesthetics or/and parecoxib
	History of chronic alcohol or opioid use
	Absolute and relative contraindications for spinal anesthesia or/and peripheral femoral nerve block, including coagulopathy, local infection, preexisting neurological disorders, or patient refusal
	Known/documented psychiatric disorders
	Pregnant or breastfeeding female
	Patient refusal to provide written informed consent

Informed written consent was provided by all patients prior to their enrolment in the study. The protocol of this study was reviewed and approved by the Institutional Review Board and the Local Ethics Committee on Human Research and Studies of Asklepieion University Hospital with the reference code #S-138/15-06-10 and following the Helsinki Declaration of 1964, revised in 2013. Each patient was assigned a code and all data were collected and analyzed in order to guarantee anonymity.

After receiving written informed consent, on the morning of surgery patients were randomly assigned into one of the two groups (D or P) using computer-generated tables and sealed drawing-coded opaque envelopes. Both study drug (parecoxib and placebo) solutions were prepared in an aseptic setting by a study nurse dedicated to the procedure. Both hospital personnel (surgeons, anesthesiologists, and nurses) and all patients were kept blind to the drug and treatment group assignment.

Anesthetic technique and study measurements

Prior to anesthesia induction, standard monitoring (ECG, SpO_2_, non-invasive blood pressure measurement at regular intervals) was applied. Sedation was facilitated by the intravenous administration of 1-2 mg of midazolam before the anesthesia was started. The operation was performed under subarachnoid anesthesia with the administration of 2.5-3.0 mL of levobupivacaine 0.5% in accordance with the patients’ somatometric characteristics, in a standard sterile fashion as per related international guidelines and recommendations. In addition, and prior to the spinal block institution, a CFNB was applied, driven by neurostimulation guidance, with the insertion of a peripheral femoral nerve catheter, for postoperative analgesia. The femoral nerve catheter was inserted after local anesthesia with lidocaine 1% (0.5 mg/kg) to ensure adequate skin and subcutaneous tissue infiltration, prior to the insertion of the peripheral nerve block needle and catheter. The needle was inserted just below the inguinal crease aiming at approximately 45 degrees cephalad. With the guidance of the neurostimulator, and as soon as the optimal motor response was achieved (patella reflex, at 0.4 mA), the CFNB catheter was threaded via the needle in a sterile manner. Its correct position was controlled with the administration of a single bolus of ropivacaine 0.75% 20 mL. After surgery completion, in the recovery room, a 200 mL pump was connected to the peripheral nerve catheter to facilitate a continuous infusion of ropivacaine 0.2% at a rate of 10 mL/h. The infusion was started as soon as signs of motor blockade regression of the subarachnoid block were clinically evident. It was maintained for 24 h.

Parecoxib 40 mg or placebo in equal volumes were administered intravenously twice per day to the patients assigned to the drug or the placebo groups, respectively. The first dose was administered 20 minutes before the end of surgery and with a duration of study, measurements equal to 48 h after this first dose.

The evening before surgery, all patients were instructed to use a 10-cm visual analog scale (VAS) for procedural pain (0=no pain at all to 10=worst pain imaginable) and got familiar with the State-Trait Anxiety Inventory (STAI) for adults [18]. From the day of surgery and onwards, VAS pain scores were measured and recorded at 4, 8, 12, 24, 36, and 48 h postsurgery. The pain scores were measured with patients in a resting position. Morphine intravenous patient-controlled analgesia (IV morphine PCA) was applied to all patients, as a rescue, in case postoperative analgesia level was inadequate (VAS pain score >3). The PCA pump contained a solution of 1 mg per mL, without basal (background) infusion rate, and lockout time of 10 or 20 min depending on the patient. The 20 min interval was applied in fragile patients, with high risk for respiratory depression, as per the hospital and departmental rules. The lockout time of 10 min was applied in the non-fragile patients. Bromage score (scale 0-3, 0=no motor blockade, and 3=complete motor blockade) was also recorded at the aforementioned time intervals. Prior to hospital discharge, approximately 36-48 h postoperatively, all patients were asked to fill in the Spielberger STAI questionnaire to evaluate anxiety levels pre- and postsurgically. Rehabilitation parameters such as range of motion (ROM) in degrees at 36 h after surgery and total length of hospital stay in days were recorded as well. Under the supervision of an anesthesiologist, blinded to the substance administered, all patients were fully conscious and in stable clinical condition at the time of the STAI questionnaire completion.

Statistical analysis

Based on a pilot study performed in our department, a 10% improvement in ROM was expected for the parecoxib group. We assumed a 7% difference between the study and control group. According to power analysis, with the support of software GPower 3.1 (Düsseldorf, Germany: University of Düsseldorf), a total of 86 patients were required to detect such a difference, with a power of 90% and a type I error (α-level) probability of 5%.

Continuous variables are presented according to their distribution as mean±SD or medians and interquartile range. Categorical variables are presented as continuous and absolute values. The independent sample t-test or the Mann-Whitney U test was used for between-group comparisons. The chi-square or Fisher’s exact test was used for pairwise comparisons of proportions and their 95% CIs were calculated. Repeated measures general linear models ANOVA and Bonferroni post hoc test were used to investigate the factor “time” between groups. The statistically significant level was set at p≤0.05. Statistical analysis of collected data was performed using the SPSS version 19.0 for Windows (Armonk, NY: IBM Corp).

## Results

**Figure 1:**
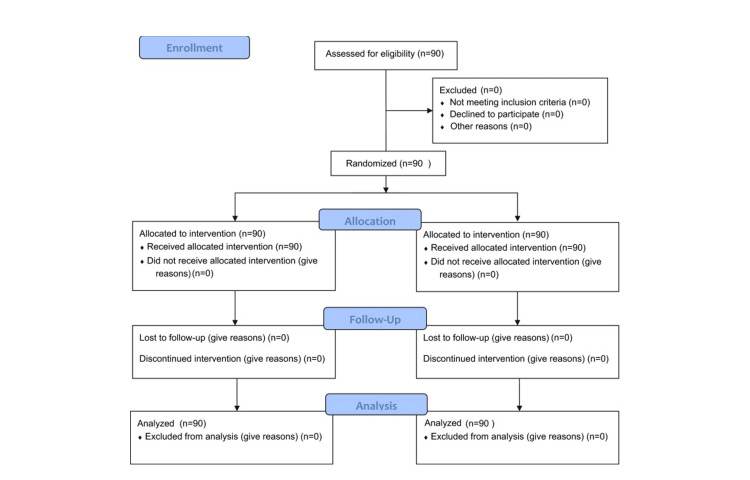
CONSORT flow diagram of the study. CONSORT: Consolidated Standards of Reporting Trials

A total of 90 patients, who completed the study protocol uneventfully, were analyzed. All patients received the appropriate intervention, and no patient was lost to follow-up. No serious complication or important adverse event has been reported in any of the patients included in the two groups. The study Consolidated Standards of Reporting Trials (CONSORT) flow diagram is presented in detail in Figure 1.

Patients’ baseline demographic characteristics as well as operative and hospitalization data did not differ between groups (Table 2). Duration of surgery and total length of hospital stay were similar between the two study groups, irrespective of the type of intervention (p>0.05).

**Table 2:** Patients' demographic, surgical and hospitalization characteristics. Group D: investigation drug (parecoxib); Group P: placebo (control)

Variables	Group D (n=45)	Group P (n=45)	p-Value
Age (years)	70.3±7.0	70.7±6.5	0.77
Gender distribution (female:male)	40:5	35:10	0.157
Weight (kg)	81.4±11.0	84.2±11.4	0.259
Height (cm)	161.9±6.0	164.9±7.0	0.13
Duration of surgery (min)	136.3±29.0	139.6±24.0	0.645
Total hospital length of stay (days)	3.71±0.9	4.07±0.9	0.102

Rehabilitation parameters are presented in Table 3. The Bromage scores did not differ between groups at any checking time point (p>0.05), whereas the ROM at 36 h after surgery was significantly improved in the group receiving parecoxib (p<0.0001).

**Table 3:** Comparison of rehabilitation parameters between the two study groups. Group D: investigation drug (parecoxib); Group P: placebo (control); ROM: range of motion

Variables	Group D (n=45)	Group P (n=45)	p-Value
Postoperative Bromage score (scale 0-3)			
4 h	2.98±0.40	2.91±0.42	0.515
8 h	1.40±0.69	1.36±0.53	
12 h	1.04±0.21	1.02±0.15	
24 h	1.0±0	1.0±0	
36 h	1.0±0	1.0±0	
Postoperative ROM (degrees)			
36 h	78.7±9.62	67.44±9.63	<0.0001

**Figure 2:**
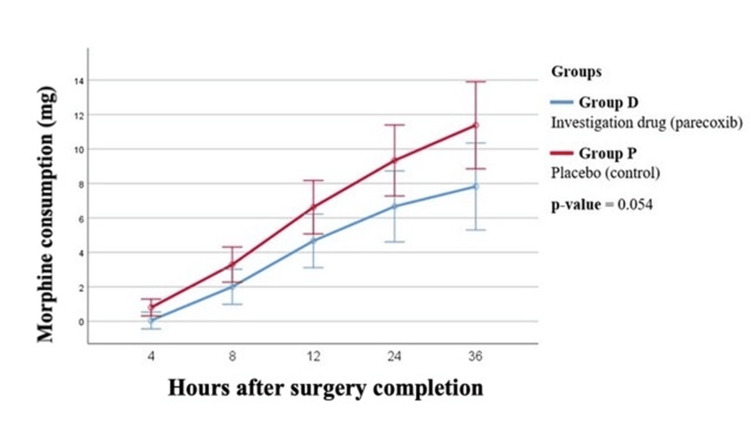
Postoperative morphine consumption via IV PCA in the two study groups. PCA: patient-controlled analgesia

**Figure 3:**
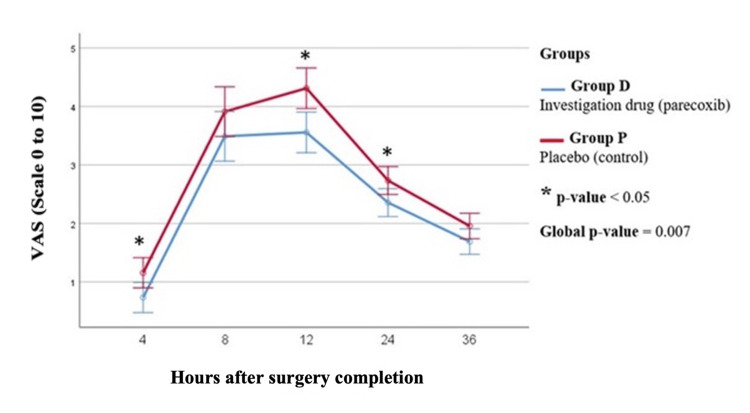
Postoperative VAS scores in the two study groups. VAS: visual analog scale

Results of postoperative analgesia levels are presented in Table 4 and in Figures 2, 3. A trend towards less morphine requirements was recorded in patients treated with parecoxib at all checking time points, corresponding to a global p-value equal to 0.054, which, even if not proven to be statistically significant, was clinically important. In addition, postoperative VAS scores for pain were significantly lower in the intervention group throughout the whole follow-up period, with more intense differences at 4, 12, and 24 h after surgery and a global p-value equal to 0.007.

**Table 4:** Comparison of postoperative analgesia levels and morphine consumption between the two study groups. Group D: investigation drug (parecoxib); Group P: placebo (control); VAS: visual analog scale; PCA: patient-controlled analgesia

Variables	Group D (n=45)	Group P (n=45)	p-Value
Postoperative VAS score (scale 0-10)			0.007
4 h	0.73±0.75	1.16±0.98	0.044
8 h	3.49±1.53	3.91±1.33	0.202
12 h	3.56±1.16	4.31±1.18	0.001
24 h	2.36±0.71	2.73±0.89	0.012
36 h	1.69±0.70	1.96±0.77	0.06
Postoperative morphine consumption via IV PCA (mg)			
Total	21.20±3.47	31.42±6.32	0.054
4 h	0.04±0.30	0.80±2.31	-
8 h	2.00±1.86	3.29±4.50	-
12 h	4.67±3.41	6.62±6.58	-
24 h	6.67±5.27	9.33±8.30	-
36 h	7.82±6.49	11.38±9.90	-

STAI scores were measured to be 39.9±5.1 in the parecoxib group and 44.1±7.7 in the placebo group (p=0.07). Interestingly, a significant correlation was found between ROM and VAS scores 12 hours after surgery (rho=-0.25, p=0.02). Also, ROM correlated inversely with STAI score postoperatively (rho=-0.2, p=0.049). Another correlation was found between postoperative STAI score and not only VAS score at 12 h (rho=0.2, p=0.03) but also VAS score at 24 h (rho=0.21, p=0.047). Morphine consumption at 4 h postoperatively was significantly associated with higher STAI scores after surgery (rho=0.215, p=0.043).

## Discussion

This randomized, double-blind, placebo-controlled study, has demonstrated that parecoxib administration, as an add-on to the postoperative analgesia protocol, is of benefit, resulting in improved ROM and lower VAS scores after surgery in patients undergoing TKA.

Peripheral nerve blocks with concomitant insertion of catheters for continuous infusion of local anesthetics with either the use of ultrasound or neurostimulators are part of common practice in terms of effective pain management [19]. The CFNB has a well-recognized analgesic efficacy in the treatment of postoperative pain after TKA, improving patients’ outcomes, by facilitating the whole rehabilitation process [20]. However, it may be associated with nerve injury in rare situations and prolonged motor blockade more frequently, complicating the mobilization of the patients [20]. Such an adverse event, which is of concern for physicians and physiotherapists, can be avoided by modifying the concentration of the administered local anesthetic or by selecting alternative peripheral blocks, such as adductor canal block (ACB), even if the CFNB has resulted in being more efficacious in this population [17]. In addition, local anesthetics infiltration (LAI) in the knee joint by the orthopedic surgeon, which is becoming very popular, is successful and nowadays frequently utilized, while patient-controlled analgesia with intravenous morphine is also successful in postoperative analgesia [21]. Nevertheless, its use is hindered by opioid adverse effects, such as nausea, vomiting, dizziness, increased percentages of postoperative delirium, and the risk of respiratory depression, often resulting in its withdrawal in certain patients’ categories [21]. Our protocol incorporated CFNB in all patients and on-demand PCA with morphine, while randomization was based on parecoxib administration.

Among all the available rehabilitation indices, perhaps the most popular and easy to estimate is ROM [22]. Our goal was to highlight any potential effect of our interventions on ROM during the first postoperative days. ROM is a valuable marker of improved mobility, as increased early postoperative ROM (days one to four) is a predictor of an increased long-term postoperative ROM [23]. In a previous study comparing patients receiving parecoxib versus placebo, no difference was reported on ROM [24]. However, peripheral nerve blocks were not part of the analgesic protocol. In another study receiving solely local analgesia, there was no difference in ROM and time-limited lower mean pain score was evident only during the first postoperative day [25]. Maximum VAS recorded was also lower for the local analgesia group. There was a remarkable difference in ROM when patients received a cocktail of local analgesics, including bupivacaine, morphine, and ketorolac, with a p<0.001. The ROM was 90 degrees in the intervention group, as opposed to the control group performing at 59-degree flexion.

Despite the superior rehabilitation profile of the parecoxib group, there was no reduction in the length of hospital stay between the two groups. On the contrary, another research group documented a significantly shorter hospitalization in the group that received bupivacaine and other analgesics as compared to the control group [26]. Furthermore, Lamplot et al. reported a similar decrease using multimodal therapy compared with opioid PCA alone [27].

Our results recorded lower pain scores for patients receiving parecoxib in compliance with the results of a previous metanalysis [28]. In addition to this, the correlation found between VAS and STAI scores is consistent with a previous study reporting reduced STAI scores in patients treated with parecoxib [29]. On the other hand, opioids with all their side effects delay rehabilitation and may increase the length of stay [30]. Regarding morphine, a trend was found towards less consumption in our sample. Similar effects of opioid-sparing effects were demonstrated also in studies using local infiltration analgesia (LIA). Morphine requirements were reduced in patients treated with combined parecoxib and LIA [31]. After TKA, LIA with ropivacaine has become a common approach for reducing postoperative pain [32]. In LIA, a high dose of the analgesic mixture is injected in the operated area either alone or combined with epinephrine, ropivacaine, and ketorolac [33]. When compared to ropivacaine alone or saline injections, the addition of ketorolac resulted in lower opioid intake, lower pain severity, and earlier hospital discharge [33].

Numerous developments, such as less painful procedures and new pain management techniques, have lately been introduced in the field of total joint arthroplasty to speed up patient recovery and reduce perioperative pain. While some preliminary evidence on the impact of smaller incisions for complete hip arthroplasty is available, further research is needed for TKA [34]. Also, the use of robotic surgery is interesting, but more studies from this perspective are necessary [35].

Looking into the Bromage score, we did not find any difference between groups. A study by Zinkus et al. compared CFNB and continuous intra-articular block (CIAB) for TKA and focused on knee function rehabilitation [36]. The study demonstrated significantly lower Bromage scores in the first 72 hours postsurgery in the CIAB group. This group had superior passive maximum ROM one month after surgery and superior active maximum ROM on day seven and one month after the surgery (p>0.05), while opioid consumption was also lower. Therefore, CIAB resulted in reduced motor block and better knee function associated with quicker rehabilitation. Looking into CFNB for TKA, the selection of the local anesthetic may also have an impact on the Bromage score and motor block. Bupivacaine 0.125% has a stronger effect on the intensity of motor block compared to ropivacaine 0.2% that we injected [37]. This aspect should definitely receive more attention from clinical pharmacologists.

A final comment is necessary in relation to the use of multimodal analgesia. Our protocol was actually an expanded multimodal analgesia, specifically caring for the intense inflammatory reaction of the tissues after such a devastating surgery. The usefulness of NSAIDs in postoperative pain management has been widely shown [10,38,39]. This perspective, randomized study in a specific surgical population has confirmed, once more, the importance of the block of cyclooxygenase cascade in postoperative pain patients.

Limitations and strengths of the study

Limitations to the generalizability of this strategy are the inherent restrictions of parecoxib use on patients with concomitant diseases who suggest avoiding it. Another limitation is the lack of fall recordings after CFNB. Moreover, we could comment on the CFNB not being executed with the support of an ultrasound machine, but at the time of the study, our anesthesia department did not have available such device. So, a further limitation of this study was represented by the fact that with the ultrasound machine, the total dose of local anesthetics to be administered at the periphery would have been reduced, potentially resulting in a reduced risk of falls in the immediate postoperative period, without jeopardizing the analgesic effect. The main advantage of the study is that its findings may be readily applied in clinical practice to improve a major factor of rehabilitation after TKA with negligible side effects.

## Conclusions

This randomized trial has shown that parecoxib is a safe and effective analgesic that may provide added benefit when combined with CFNB. It can improve the rehabilitation parameters in postoperative TKA patients and the overall pain management plan. The added benefits were certainly related to its activity on the inflammatory reactions consequence of the devastating surgery. Moreover, it has been reconfirmed that multimodal analgesia is always the best solution to have the best acute pain management after surgery. This analgesia modality provides a comfortable life quality to the patient, allows the possibility of good rehabilitation, and helps to prevent acute pain chronification.
